# Fights on the surface prior to fungal invasion of insects

**DOI:** 10.1371/journal.ppat.1011994

**Published:** 2024-02-22

**Authors:** Junmei Shang, Song Hong, Chengshu Wang

**Affiliations:** 1 Key Laboratory of Insect Developmental and Evolutionary Biology, CAS Center for Excellence in Molecular Plant Sciences, Shanghai Institute of Plant Physiology and Ecology, Chinese Academy of Sciences, Shanghai, China; 2 CAS Center for Excellence in Biotic Interactions, University of Chinese Academy of Sciences, Beijing, China; 3 School of Life Science and Technology, ShanghaiTech University, Shanghai, China; University of Maryland, Baltimore, UNITED STATES

## Abstract

Entomopathogenic fungi (EPF) infect insects by landing on and penetrating cuticles. Emerging evidence has shown that, prior to the invasion of insects, fungal cells have to battle and overcome diverse challenges, including the host behavioral defenses, colonization resistance mediated by ectomicrobiotas, host recognition, and generation of enough penetration pressure. The ascomycete EPF such as *Metarhizium* and *Beauveria* can thus produce adhesive proteins and/or the exopolysaccharide mucilage to tightly glue fungal cells on cuticles. Producing antimicrobial peptides and chemical compounds can enable EPF to outcompete cuticular defensive microbes. The use of divergent membrane receptors, accumulation, and quick degradation of lipid droplets in conidial cells can help EPF recognize proper hosts and build up cellular turgor to breach cuticles for systematic invasion. Further investigations are still required to unveil the multifaceted and intricate relationships between EPF and insect hosts.

## Introduction

Entomopathogenic fungi (EPF) infect insects by landing on and invading via penetration of host cuticles, the critical barriers that EPF must recognize and breach to enter body cavities [1]. By using the ascomycete EPF such as the *Metarhizium* and *Beauveria* species as models, extensive studies have revealed the proteins and enzymes involved in degrading the protein- and chitin-rich insect cuticles [2]. However, insect hosts like flies, bees, termites, and ants have evolved hygienic behaviors that can detect and clear off the spores of EPF before fungal initiation of invasion [3]. Locusts, however, can mediate resistance to *Metarhizium* infection by raising body temperature [4]. In addition, similar to humans and plants [5,6], individual insects have been appreciated as holobionts, the hosts that house and live with diverse ecto- and endosymbiotic microbes [7,8]. It has been shown that insect cuticular bacteria can mediate colonization resistance against fungal parasite infections [9,10]. Here, we summarize the advances of insect defense and EPF counterdefense on insect body surfaces before fungal penetration and invasion of hosts.

## 1. EPF fight with insect hygienic grooming

**Fig 1:**
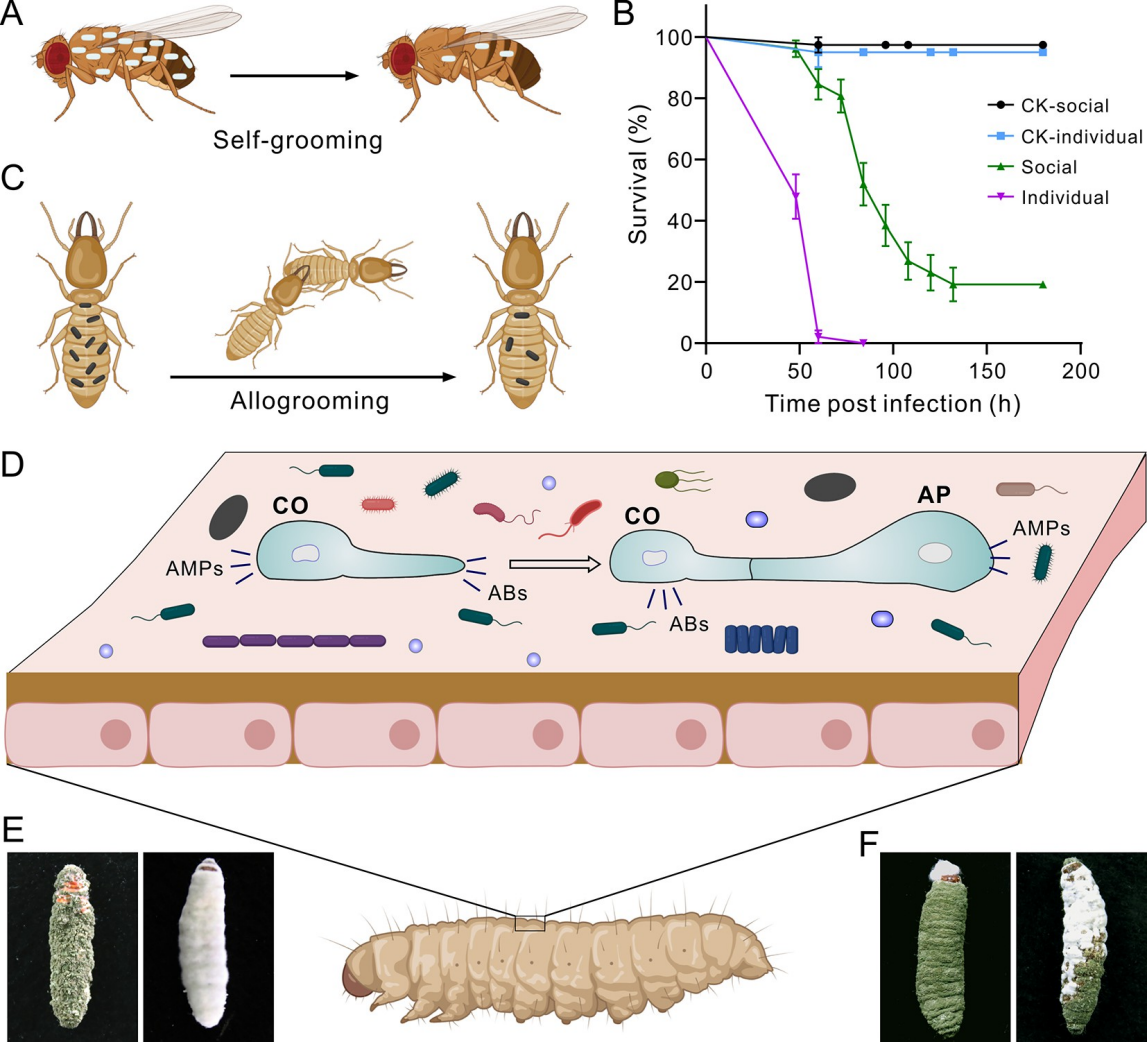
Illumination of fungal fights on insect body surfaces for host infection. (**A**) Illuminated removal of fungal spores off the *Drosophila* body surface via self-grooming. (**B**) Divergent survival of termite (*C*. *formosanus*) workers after the topical infection with the spore suspensions of *M*. *robertsii* (5 × 10^5^ conidial/ml in 0.05% Tween 20) and being placed in different forms. Social: the treated termites were placed in groups (10 per Petri dish in 6 cm diameter); individual: the treated termites were individually placed in dishes (one per dish). The termites treated with the Tween and placed either socially or individually were used as CKs. (**C**) Illuminated removal of fungal spores off the termite body surface via allogrooming. (**D**) Schematic of the *Metarhizium* spore responses against cuticular microbes on insect body surface. Divers bacterial cells are cartooned, while the big-sized cells represent other fungal cells. (**E**) The cadavers of the wax moth larvae killed and uniformly mycosed by Mr (left) and Bb (right) after individual infections. (**F**) The representative cadavers of the wax moth larvae killed and mycosed by both Mr and Bb after topical infection with the mixed spore suspension (at a ratio of spores: Mr:Bb = 1:9) [26]. Panels A, C, and D were created with BioRender.com and not to scale. Panel B was generated based on our bioassay data using the program GraphPad. Images shown in Panels E and F were taken during our previous study [26]. ABs, antibiotics; Bb, *B*. *bassiana*; AMPs, antimicrobial peptides; AP, appressorium; CKs, controls; CO, conidium; Mr, *M. robertsii*.

During the arms race coevolution between EPF and insects, some EPF species evolved the ability to maneuver host behavior to facilitate the dispersal of fungal propagules [11]. Different insects, however, especially flies and eusocial insects such as ants, termites, and bees, have evolved behavioral or social immunity to defend hosts against parasites by hygienic grooming or social distancing [12]. After topical application to *Drosophila melanogaster*, more than 70% of *Metarhizium robertsii* spores could be cleared off by flies through self-grooming within a few hours postinoculation (Fig 1A). Interestingly, *D*. *melanogaster* adults can cognitively detect a spore surface protein of *M*. *robertsii* through a chemosensory protein, and this promptly triggers fly hygienic behavior [13].

Following immersion of the Formosan subterranean termites (*Coptotermes formosanus*) in the spore suspension of *M*. *robertsii*, treated insects were divided either individually or socially (every 10 insects) into Petri dishes. Interestingly, lone termites died much more quickly than those grouped with nestmates (Fig 1B). Similar to the previous observation [14], grouped termites could help each other to remove fungal spores through allogrooming (Fig 1C). To counterattack the host hygienic removals, the spores of *Metarhizium* and *Beauveria* secrete adhesin proteins to facilitate spore adherence to insect cuticles [15,16]. *Metarhizium* species can also produce the adhesive exopolysaccharide mucilage to fortify cell adhesion upon spore germination and appressorium formation [17]. Otherwise, spore landing and “hiding” on the intersegmental regions or spiracles of insects can better escape host behavioral clearance [13].

### 2. Outcompetition of insect cuticular bacteria by EPF

It is now known that insect cuticles are inhabited by diverse ectomicrobiotas [18] (Fig 1D). EPF must pave the way for success in fending off defensive bacteria. Similar to the findings in animals and plants, antimicrobial peptides (AMPs) are also encoded by fungi [19]. For example, a defensin-like BbAMP1 encoded by *B*. *bassiana* can enable the fungus to suppress the cuticular protective bacteria [20]. Intriguingly, the *BbAMP1*-like gene is absent in *Metarhizium* species. Instead, the potent antibiotic helvolic acid, a nortriterpenoid, produced and accumulated in the spores of *M*. *robertsii* can help the fungus outcompete insect cuticular bacteria [21]. Indeed, EPF can produce different compounds with antibiotic activities [22]. A super biosynthetic gene cluster of *M*. *robertsii* produces 4 classes of antibiotic compounds that function as a synergistic cocktail against diverse bacteria, including those isolated from insect cuticles [23]. Otherwise, the iron chelators produced by EPF such as *B*. *bassiana* are implicated in mediating competitive deprivation of micronutrients to inhibit microbes in diverse microniches, including insect cuticles [24]. Overall, EPF have evolved multiple ways to suppress defensive bacteria inhabiting insect cuticles (Fig 1D).

## 3. Competitive exclusion among EPF and between EFP and other fungi on the host surface

In addition to bacteria, insect cuticles may be inhabited by different transient or resident fungal cells (Fig 1D). In particular, EPF are ubiquitously present and the spores of different species or strains may simultaneously or sequentially land on the cuticle of individual insects to theoretically initiate coinfections. However, the insects freshly killed by EPF in the field are typically colonized and mycosed by a single species or strain [25] (Fig 1E), suggesting that the killing and co-colonizing a single insect by 2 fungi or more might unlikely take place in nature. The laboratory coinfection of the wax moth (*Galleria mellonella*) larvae with the different ratios of the *M*. *robertsii* (Mr) and *B*. *bassiana* (Bb) spores revealed that, similar to animals, the phenomenon of competitive exclusion (CR) occurred between 2 fungi when competing for individual insects. The former could suppress the infection and colonization of individual caterpillars by *B*. *bassiana* even when being used in a 1:9 (Mr:Bb) ratio of spore numbers [26]. Under this condition, only a few of the cadavers were colonized by 2 fungi (Fig 1F). The occurrence and effect of CR among different EPF in the field require further investigation.

Many insect herbivores are also the vectors of plant diseases, including phytopathogenic fungi. For example, different *Fusarium* species have been frequently found as the mutualists of beetles and caterpillars, which benefits disease transmission [27]. The mycobiome analysis of the tortoise beetle *Chelymorpha alternans* revealed that this insect can be inhabited by *Fusarium oxysporum* throughout its lifecycle, including the conspicuous coating of beetle pupae by the fungus to confer defense against predators [28]. In this respect, CR against other fungi may also be a prerequisite for EPF to invade insects. It has been shown that the endophytic *Metarhizium* species have the potency against *Fusarium* diseases [29]. Similar to the secretion of antimicrobials against defensive bacteria, producing antifungal peptides and secondary metabolites such as the cyclopeptides by EPF may benefit EPF to outcompete other fungi [30,31], suggesting that EPF must have evolved the tolerant and self-protection tactics against these antifungal components.

## 4. EPF recognition of insect hosts

Phylogeny analysis has shown that the ascomycete EPF such as *Metarhizium* and *Beauveria* species exhibit a closer evolutionary relationship to phytopathogens than to mammalian pathogenic fungi [32]. For example, *Metarhizium* diverged shortly after *Fusarium* and evolved dozens of species with divergent host specificity [33]. Given the diversity of insect species and their cuticles, EPF have to properly recognize insect hosts for invasion. Cytoplasm membrane receptors have been unveiled in mediating host and environmental sensing [34]. The loss-of-function investigation of divergent G-protein coupled receptors (GPCRs) in the generalist *M*. *robertsii* revealed that there might be a core GPCR involved in recognizing all examined insect cuticles while other GPCRs jointly or individually contribute to the detection of specific insect hosts [35]. Nevertheless, the host-specific ligands detected by EPF receptors remain elusive. It is noteworthy that EPF have also evolved the enzymes to detoxify the toxic cuticular components of some insects such as the tenebrionid beetles before cuticle penetration [36].

## 5. EPF generation of penetration turgor

In addition to the secretion of cuticular degrading and detoxifying enzymes, the last step in EPF invasion of insects requires the accumulation of cellular turgor pressure for cuticle penetration [2]. The lipid droplets (LDs) within the *Metarhizium* conidial mother cells will be transferred into the infection structure appressoria, and the autophagic components will then mediate the internalization of LDs into vacuoles for quick degradation into the high concentration of glycerol via a microlipophagy machinery [37]. Similar to some plant-pathogenic fungi like *Fusarium* species, some EPF like *Beauveria* species do not produce appressoria for cuticle penetration. However, the autophagic genes of *B*. *bassiana* are similarly required for cuticle penetration and, therefore, fungal virulence against insects [38]. Different insect species ranging from the rigid beetles to soft-bodied caterpillar larvae have disparate rigidity of exoskeletons. It remains elusive how EPF, especially those generalist species, can finely tune the cellular turgor pressure to pierce diverse insect cuticles.

## Outlook

It has been overlooked until recently that fungal infection of insects has to outwit host-defensive ectomicrobiotas before cuticle penetration and invasion of hosts. These findings will expand the future investigation of the bilateral fungus–insect interactions to multipartite relationships among EPF, ectomicrobiotas, and insect hosts. Future efforts are also required to better understand fungal tactics to combat insect behavioral defenses, correctly sense and recognize diverse insect hosts, and quickly generate cellular turgor to penetrate different host cuticles. The data of these studies may benefit the better use of EPF against insect pests while the protection of beneficial insects like bees and silkworms using probiotic bacteria.
